# ASSET-BASED COMMUNITY DEVELOPMENT FOR DEMENTIA-FRIENDLY COMMUNITIES (ABCD FOR DFC): A PILOT PROJECT IN HONG KONG

**DOI:** 10.1093/geroni/igad104.1608

**Published:** 2023-12-21

**Authors:** Cheryl Chui, Shiyu Lu, On Fung Chan, Terry Lum

**Affiliations:** The University of Hong Kong, Hong Kong, Hong Kong; City University of Hong Kong, Kowloon, Hong Kong; The University of Hong Kong, Hong Kong, Hong Kong; The University of Hong Kong, Hong Kong, Hong Kong

## Abstract

There is little understanding about how a dementia-friendly community can be constructed in East Asian societies, particularly in geographically remote areas characterized with majority older adult residents and limited external support. A pilot project using an asset-based community development (ABCD) approach in constructing a dementia-friendly community in Hong Kong was implemented. Informed by the theoretical tenets of ABCD, the purpose of this paper is to examine the effects of this pilot project in enabling older adult residents and other community stakeholders to better respond to challenges resulting from growing prevalence of dementia. Multiple stakeholder interviews were conducted with older adult residents, church leaders, district councillors, and social workers. Participants were asked to share the strengths and challenges of this ABCD pilot project in building a dementia-friendly community. Data generated from interviews were analyzed using thematic analysis. Four key preliminary themes were identified, including on the individual level: enhanced dementia literacy, and enacted proactive behaviour; and on the community level: strengthened bridging social capital, and improved community caring capacity. Uncertain long-term financial and professional support posed challenges to participants. Nevertheless, findings underscore the importance of departing from traditional service delivery models, to one that infuses ABCD approaches in constructing a dementia-friendly community. Mobilising existing human and social capital in the community, and embedding older adults in co-creating solutions are key to improve dementia-related interventions. We contribute to ongoing theoretical and practice discussions on the intersections between population aging and employing community-based solutions to create a more dementia-friendly society.

